# Direct Anterior Approach in Lateral Decubitus Position Versus Supine Position for Unilateral Total Hip Arthroplasty: A Comparative Study

**DOI:** 10.1111/os.12881

**Published:** 2021-03-11

**Authors:** Wenbo Zhao, Shanwu Li, Yi Yin, Zhiqiang Wang, Guanjun Sun, Xu Peng, Jiaping Lan, Yongjie Ye

**Affiliations:** ^1^ Department of Joint Surgery Suining Central Hospital Suining China

**Keywords:** Arthroplasty, Hip, Lateral decubitus position, Replacement, Supine position

## Abstract

**Objective:**

To compare the clinical efficacy of the direct anterior approach in lateral decubitus position (L‐DAA) and supine position (S‐DAA) for unilateral total hip arthroplasty.

**Methods:**

A retrospective study was conducted on 89 patients who underwent primary unilateral total hip arthroplasty in our department between August 2016 and December 2017. There were 46 patients who underwent L‐DAA and 43 patients who underwent S‐DAA. The body mass index (BMI), operation time, blood loss, preoperative Hb, first day and third day postoperative Hb, incision length, hospital stay, preoperative and postoperative Harris score, preoperative and postoperative visual analogue scale (VAS) score, radiological evaluation, intraoperative and postoperative complication, postoperative absolute length difference of lower extremity were recorded and analyzed. *P* < 0.05 was set as the significant difference.

**Results:**

All patients were followed up for 8–23 months, with an average of 15.6 months. No significant differences were found in preoperative and postoperative Harris scores, preoperative Hb, incision lengths, radiological evaluations, preoperative and postoperative VAS scores, and hospital stay (*P* > 0.05). However, significant differences were detected in BMI, blood loss, first day and third day postoperative Hb, and operation time (*P* < 0.05). There were no postoperative complications in the L‐DAA and S‐DAA groups. During the operation, two cases of proximal femoral fracture occurred in the L‐DAA group, four in the S‐DAA group, and the difference was statistically significant. There were significant differences found in the postoperative absolute length difference of lower extremity between the two groups.

**Conclusion:**

Compared with the S‐DAA approach, the L‐DAA approach had the advantages of shorter operation time and less blood loss. Compared with S‐DAA, it was easier to expose the proximal femur, and lower BMI was required in L‐DAA. However, it was more difficult to compare the length of both lower extremities in the L‐DAA approach than in the S‐DAA approach.

## Graphical abstract

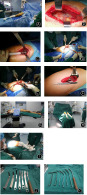

Lateral decubitus position: A. Surgical position; B. hemostasis of the extrafemoral artery achieved; C. Grinding the acetabulum; D. Position of operation in femur and acetabulum; E. Reaming the femur and acetabulum; F. Length of incision. Supine position: G. Surgical position; H. Skin preparation and draping; I. Position of operation in femoral; J & K. Muscle protection retractors.

## Introduction

With an increasing number of patients with end‐stage hip disease, total hip arthroplasty (THA) is being widely used^1^. Traditional THA often adopts the posterolateral approach (PLA) or direct lateral approach. In recent years, along with the idea of Enhanced Recovery After Surgery (ERAS), the direct anterior approach (DAA) has been accepted by orthopedists^2^. DAA can expose the hip joint through the gap between the tensor fasciae latae and sartorius and avoid injuring the extorsion muscle^3^. Depending on the surgical position, DAA can be classified as direct anterior approach in lateral decubitus position (L‐DAA) and direct anterior approach in supine position (S‐DAA). Compared with the traditional operation, S‐DAA requires a special operating bed and has unique complications, which limit the clinical application of this operation. There is an obvious absence of literature that compares the clinical efficacy of the L‐DAA with S‐DAA for unilateral total hip arthroplasty. However, there are a few studies that compare these two surgical approaches. We conducted a retrospective study to analyze 89 patients who had been treated with unilateral total hip replacement in our department from August 2016 to December 2017, comparing the clinical efficacy of the direct anterior approach in lateral decubitus position (L‐DAA) and supine position (S‐DAA) for unilateral total hip arthroplasty.

## Materials and Methods

There were a total of 89 patients in our study. According to the difference in surgical approach, all patients had been divided into L‐DAA group and S‐DAA group. Informed consent was obtained from all patients. Inclusion criteria: (i) unilateral end stage hip diseases; (ii) primary replacement; (iii) no history of surgery in the affected hip; and (iv) complete follow‐up data. Exclusion criteria: (i) bilateral surgery; (ii) type Crowe IV developmental hip dysplasia; (iii) proximal femur deformity; (iv) stiff hips; and (v) patients with severe osteoporosis.

### Surgical Methods

Surgical plan was made based on the DR and CT preoperatively. Intravenous antibiotics were used half an hour prior to surgery. Intravenous tranexamic acid was used before skin incision. All patients were administered general anesthesia. The hip prosthesis was supplied by LINK Corporation in Germany and the muscle protection retractor had been prepared preoperatively.

#### Anesthesia and Position

All patients were administered general anesthesia. L‐DAA in lateral position and S‐DAA in supine position.

#### Aapproach and Exposure

Based on the anterior superior iliac crest and fibula head, a direct incision was made 2 cm below the anterior superior iliac crest and extended to the fibula head. The incision was about 8–12 cm long. We separated the tensor fasciae latae and sartorius through the Hueter gap and exposed the extrafemoral artery. We achieved hemostasis of the artery and used three muscle protection retractors placed on the upper and lower sides of the femoral neck and lateral aspect of the greater trochanter. We made a “T” incision in front of the joint capsule and removed the anterior glenoid labrum if the inflammatory response was significant. Next, we performed first osteotomy below the femoral head and second osteotomy in the femoral neck based on the template. We then removed the bone fragments and placed two acetabular retractors at the 4 and 8 o'clock positions. The assistant maintained proper traction of the lower limb so that we could clean up the hyperplasia of the glenoid labrum and residual ligament.

#### Fixation or Placement of Prosthesis

L‐DAA: Locating the acetabular rotation center based on the transverse ligament and oval fossa, we ground the acetabulum at 40° of abduction and 15° of anteversion. We then placed the acetabular prosthesis. Next, we extended, adducted, and extorted the hip joint, placed one retractor under the femoral neck and another retractor on the abductor muscle, and loosened the joint capsule to expose the proximal femur. We then opened and reamed the femur, placed the femoral prosthesis, and performed reduction of the hip joint. Finally, we flushed and sutured the wound.

S‐DAA: The incision selection, surgical approach, and operation on the acetabulum were the same as with L‐DAA. Operating on the femur, we first placed the lower limb that was being operated upon under the other lower limb and adjusted the distal end of the operation table to 30°. Second, we maintained the hip joint extension, adduction, and extorsion, and used the retractor to expose the proximal femur. Third, we opened and reamed the femur, placed the femoral prosthesis, and performed reduction of the hip joint. Finally, we flushed and sutured the wound.

#### Postoperative Management

Antibiotics were used for 24–48 h postoperatively. Rivaroxaban was used to prevent deep vein thrombosis. Functional training was begun post‐anesthesia period.

### Outcome Measures

#### Visual Analogue Scale (VAS)

The VAS is the most commonly used questionnaire for quantification of pain. It is a continuous scale comprised of a horizontal or vertical line, usually 10 cm in length. For pain intensity, the scale is most commonly anchored by “no pain” (score of 0) and “pain as bad as it could be” (score of 10). A score of 0 is considered as no pain, 1–3 mild pain, 4–6 moderate pain, and 7–10 severe pain. We assessed the preoperative and postoperative VAS scores separately.

#### Harris Hip Score (HHS)

The HHS was used to evaluate postoperative recovery of hip function in an adult population. The HHS score system mainly includes the four aspects of pain, function, absence of deformity, and range of motion. The score standard had a maximum of 100 points (best possible outcome). A total score <70 is considered a poor score, 70–80 is fair, 80–90 is good, and 90–100 is excellent. We also assessed the preoperative and postoperative HHS separately.

In order to compare the damage of the two methods to the patients and the early postoperative recovery of the patients, we observed, recorded and analyzed the following relevant indicators: the BMI, operation time, blood loss, preoperative Hb, first day and third day postoperative Hb, incision length, hospital stay, radiological evaluations, intraoperative and postoperative complications, and postoperative absolute length difference of lower extremity. Radiological evaluation postoperatively was based on the DR and CT. The standard of acetabular abduction angle, acetabular anteversion angle, and femoral prosthesis pronation were 30°–50°, 5°–25°, and −4° to 4°, respectively^4^, ^5^, ^6^. Blood loss was calculated according to the formula proposed by Nadler *et al*.^7^.

### Statistical Analyses

SPSS 20.0 software (IBM Corporation, Chicago, USA) was adapted to analyze the data. The measurement data were expressed as the mean ± standard deviation and used the independent sample *t*‐test. The enumeration data were expressed as the case number or % and used χ^2^ test. The rank sum test was used for comparison between groups. *P* < 0.05 was considered to be statistically significant.

## Results

### Patient

All patients were followed‐up for 8–23 months, with an average of 15.6 months.

### General Results

No significant differences were found in preoperative and postoperative Harris scores, preoperative Hb, incision lengths, radiological evaluations, preoperative and postoperative VAS scores, and hospital stay (*P* > 0.05). However, there were significant differences detected in BMI, blood loss, first day and third day postoperative Hb, and operation time (P < 0.05). There were significant differences found in the postoperative absolute length difference of lower extremity between the two groups (Tables 1, 2, 3, 4, 5).

**TABLE 1:** General data between the two groups

Groups	Age (Year)	Gender (M)	BMI	HHS score preoperative	VAS score preoperative
L‐DAA(n = 46)	72.3 ± 10.8	24 (52.2%)	29.8 ± 3.4	35.1 ± 8.6	6.5 ± 1.1
S‐DAA(n = 43)	71.9 ± 12.1	22 (51.2%)	24.7 ± 2.5	35.5 ± 9.3	6.6 ± 1.4
*t* or χ^2^	0.539	0.437	0.416	0.407	0.543
*P*	>0.05	>0.05	<0.05	>0.05	>0.05

**TABLE 2:** Perioperative period between the two groups

Groups	Operation time (minutes)	Incision length (cm)	Blood loss	Hb Preoperative (g/L)	Hb first day postoperative (g/L)	Hb third day postoperative (g/L)
L‐DAA(n = 46)	60.9 ± 10.3	10.3 ± 2.1	100.4 ± 7.5	127.3 ± 11.7	119.3 ± 9.2	103.1 ± 9.1
S‐DAA(n = 43)	69.1 ± 9.7	10.1 ± 2.7	110.2 ± 8.6	125.5 ± 13.1	112.7 ± 10.3	94.2 ± 8.3
*t*	0.581	0.776	0.458	0.405	0.539	0.658
*P*	<0.05	>0.05	<0.05	>0.05	<0.05	<0.05

**TABLE 3:** Postoperative Harris and VAS scores between the two groups

Groups	Harris score			VAS score	
	7 Days postoperatively	1 Month postoperatively	6 Months postoperatively	3 Days postoperatively	7 Days postoperetively
L‐DAA(n = 46)	81.8 ± 9.2	91.2 ± 9.1	94.7 ± 9.5	1.2 ± 0.1	1.1 ± 0.1
S‐DAA(n = 43)	82.1 ± 9.1	90.1 ± 10.2	93.9 ± 9.8	1.1 ± 0.2	1.2 ± 0.2
*t*	0.626	0.682	0.639	0.445	0.467
*P*	>0.05	>0.05	>0.05	>0.05	>0.05

**TABLE 4:** Radiological evaluation postoperative between two groups

Groups	Acetabular abduction angle (°)	Acetabular anteversion angle (°)	Femoral prosthesis pronation
L‐DAA(n = 46)	15.9 ± 5.3	42.5 ± 3.1	45/46
S‐DAA(n = 43)	16.7 ± 4.2	43.7 ± 4.4	42/43
*t* or *X^2^*	1.213	0.797	0.454
*P*	>0.05	>0.05	>0.05

**TABLE 5:** Absolute length difference of lower extremity postoperatively and hospital stay between the two groups

Groups	Absolute length difference of lower extremity postoperatively(cm)	Hospital stay(days)
L‐DAA(n = 46)	1.6 ± 0.3	6.7 ± 1.2
S‐DAA(n = 43)	0.8 ± 0.2	7.2 ± 1.4
*t*	0.244	0.436
*P*	<0.05	>0.05

### Complications

There were no postoperative complications in the L‐DAA and S‐DAA groups. During the operation, two cases of proximal femoral fracture occurred in the L‐DAA group, four cases in the S‐DAA group, and the difference was statistically significant (Table 6).

**TABLE 6:** Intraoperative and postoperative complication rate between the two groups

Groups	Fracture of proximal femur (Intraoperative)	Artificial joint dislocation (Postoperative)
L‐DAA(n = 46)	2	0
S‐DAA(n = 43)	4	0
*X^2^*	0.649	0.341
*P*	<0.05	>0.05

## Discussion

With the development of artificial hip replacement technology, the choice of surgical approach had been the focus of debate among clinicians^8^. In 1881, Carl Hueter, the German orthopedist, introduced the direct anterior approach for total hip arthroplasty for the first time. After more than 100 years of development, DAA is considered one of the most suitable minimally invasive approaches of ERAS^9^, ^10^, ^11^. DAA can be classified into L‐DAA and S‐DAA. Which of the two approaches is better still remains controversial.

### L‐DAA Vs S‐DAA

Our study retrospectively analyzed the data between the two groups. We found that there was no significant differences in the Hb, Harris scores, and VAS scores preoperatively. No significant differences were found in the length of the incision, Harris scores, and VAS scores postoperatively, and hospital stay. Therefore, we concluded that DAA in both positions could result in satisfactory clinical curative effect and rapid recovery.

Because of differences in the difficulty level while operating on the femur, a significant difference was found in the operation times between the two groups. In L‐DAA, the exposure of proximal femur was easier. Therefore, the operation time was shorter. In S‐DAA, prolonged operation time could lead to more blood loss intraoperatively. Our study showed that there were significant differences between the two groups in Hb first day postoperatively and third day postoperatively. We thought the reason was that the S‐DAA group could not fully extend the lower limb after the exposure of proximal femur, and therefore the operation time was prolonged with greater blood loss. In contrast, the L‐DAA group could extend the lower limb beyond 30°, and therefore the proximal femur could be entirely exposed and reduce the incidence of proximal femoral fracture. This shortened the so‐called learning curve at the same time^12^. In conclusion, if the patient had severe osteoporosis or could not fully extend, adduct, or extort the lower limb, the incidence rate of proximal femoral fracture would get higher. In our study, there were two cases of proximal femoral fracture that occurred in the L‐DAA group and four cases in the S‐DAA group. We thought the reason was that the acetabular operation in L‐DAA was similar to the operation in posterior lateral approach, which was more familiar to the surgeon. In addition, there was no significant difference found on radiological evaluation and hip function. It was suggested that both approaches could achieve good placement of prosthesis, which was similar to the results obtained by Barrett *et al*.^13^.

For obese or strong patients in S‐DAA, it was difficult to fully expose the surgical field because of the muscle or fat tissue accumulated around the incision. In the L‐DAA group, because of gravity, the muscle or fat tissue would be pushed away from the incision, making exposure of the surgical field easier compared to S‐DAA. In our study, the BMI in S‐DAA was lower than in L‐DAA, and the difference was significant. In conclusion, thin patients with low BMI should be treated with S‐DAA and obese patients should be treated with L‐DAA. We believe that obesity is not restricted to L‐DAA, but patients with severe osteoporosis or having difficulty in extending the lower limb should select the DAA carefully.

Dislocation of total hip arthroplasty (THA) is a serious postoperative complication. Not only does it affect hip function, but it is also more likely to require revision surgery. Some authors concluded that dislocation of THA was related to the tension from surrounding soft tissue, the integrity of abductor muscle, and the placement of prosthesis^14^. DAA is applied in the neuromuscular space with no damage to muscle or posterior structure of the hip joint. Therefore, the stability of the hip joint is greatly enhanced and postoperative activities are not limited. In our study, no artificial joint dislocation was observed in both groups, which was consistent with the conclusion of the above studies.

There were significant differences found in the postoperative absolute length difference of the lower extremity between the two groups. Because the pelvic position was fixed in S‐DAA and both lower limbs could be disinfected at the same time, the surgeons could directly compare the length of both lower limbs, which led to accurate results. The length of the lower limbs was measured by fluoroscopy in L‐DAA based on the height of the lesser trochanter and ischial tuberosity or the height of the greater trochanter and acetabular rotation center of the hip joint; some cases depended on the experience of the surgeon. Therefore, the accuracy was relatively low in L‐DAA, which led to a significant difference in the postoperative absolute length difference of the lower extremity.

### Limitations of the Study

There are several limitations in our study. The research is based on a retrospective clinical evaluation with a relatively small sample size. Secondly, the mean follow‐up time of the present study was only 15.6 months, thus long‐term outcomes could not be obtained in the current study.

### Conclusion

L‐DAA had the following advantages over S‐DAA: (i) operation table did not need any adjustment; (ii) surgical instruments did not require eccentricity distance; (iii) it was easier to expose the proximal femur; and (iv) less restrictions concerning patient BMI. However, L‐DAA was not conductive to the comparison of the length of the lower limb. If bilateral surgery is needed, the patient's position and disinfection should be rearranged in the operation.
